# A comprehensive overview of urogenital, anorectal and oropharyngeal *Neisseria gonorrhoeae* testing and diagnoses among different STI care providers: a cross-sectional study

**DOI:** 10.1186/s12879-017-2402-0

**Published:** 2017-04-20

**Authors:** Casper D. J. den Heijer, Christian J. P. A. Hoebe, Geneviève A. F. S. van Liere, Jan E. A. M. van Bergen, Jochen W. L. Cals, Frans S. Stals, Nicole H. T. M. Dukers-Muijrers

**Affiliations:** 1 Department of Medical Microbiology, Maastricht University Medical Centre, CAPHRI School of Public Health and Primary Care, Maastricht, the Netherlands; 2 Department of Sexual Health, Infectious Diseases and Environmental Health, Public Health Service South Limburg, Geleenbeeklaan 2, 6166 GR Geleen, the Netherlands; 3 Department of General Practice, University of Amsterdam, Amsterdam, the Netherlands; 4 Soa Aids Nederland, Amsterdam, the Netherlands; 5 Department of Family Medicine, CAPHRI School for Public Health and Primary Care, Maastricht University, Maastricht, the Netherlands; 6 Department of Medical Microbiology, Zuyderland Medical Centre, Heerlen, the Netherlands

## Abstract

**Background:**

Gonorrhoea, caused by *Neisseria gonorrhoeae* (NG), can cause reproductive morbidity, is increasingly becoming resistant to antibiotics and is frequently asymptomatic, which shows the essential role of NG test practice. In this study we wanted to compare NG diagnostic testing procedures between different STI care providers serving a defined geographic Dutch region (280,000 inhabitants).

**Methods:**

Data on laboratory testing and diagnosis of urogenital and extragenital (i.e. anorectal and oropharyngeal) NG were retrieved from general practitioners (GPs), an STI clinic, and gynaecologists (2006–2010). Per provider, we assessed their contribution regarding the total number of tests performed and type of populations tested, the proportion of NG positives re-tested (3–12 months after treatment) and test-of-cure (TOC, within 3 months post treatment).

**Results:**

Overall, 17,702 NG tests (48.7% STI clinic, 38.2% GPs, 13.1% gynaecologists) were performed during 15,458 patient visits. From this total number of tests, 2257 (12.7%) were extragenital, of which 99.4% were performed by the STI clinic. Men were mostly tested at the STI clinic (71%) and women by their GP (43%).

NG positivity per visit was 1.6%; GP 1.9% (*n* = 111), STI clinic 1.7% (*n* = 131) and gynaecology 0.2% (*n* = 5). NG positivity was associated with *Chlamydia trachomatis* positivity (OR: 2.06, 95% confidence interval: 1.46–2.92).

Per anatomical location, the proportion of NG positives re-tested were: urogenital 20.3% (*n* = 36), anorectal 43.6% (*n* = 17) and oropharyngeal 57.1% (*n* = 20). NG positivity among re-tests was 16.9%.

Proportions of NG positives with TOC by anatomical location were: urogenital 10.2% (*n* = 18), anorectal 17.9% (*n* = 7) and oropharyngeal 17.1% (*n* = 6).

**Conclusions:**

To achieve best practice in relation to NG testing, we recommend that: 1) GPs test at extragenital sites, especially men who have sex with men (MSM), 2) all care providers consider re-testing 3 to 12 months after NG diagnosis and 3) TOC is performed following oropharyngeal NG diagnosis in settings which provide services to higher-risk men and women (such as STI clinics).

## Background

Gonorrhoea, caused by the Gram-negative diplococcus *Neisseria gonorrhoeae* (NG), is a bacterial sexually transmitted infection (STI) which causes reproductive morbidity, can facilitate human immunodeficiency virus (HIV) transmission, and is increasingly becoming resistant to antibiotics [1]. The high number of asymptomatic episodes in urogenital (in women) and extragenital (in men and in women, i.e. anorectal and oropharyngeal) NG highlights the essential role of NG test practice by STI care providers [2, 3].

NG testing and treatment should be targeted towards at-risk groups, the ‘key populations’, in order to achieve efficient NG control. Determinants that have been associated with increased NG positivity are young age, low socioeconomic status, men who have sex with men (MSM) and a previous positive NG test [1, 4]. Additionally, NG is associated with other genital mucosal pathogens, notably *Chlamydia trachomatis* (CT) [5]. Identification of these determinants can help to detect potentially hidden, under-tested key populations and optimise NG control.

In the Netherlands, STI clinics and general practitioners (GPs) (i.e. public health care) have a major role in STI care, comparable to that provided in the United Kingdom [6] and Australia [7]. Dutch STI clinics serve specific high-risk groups, including young people (aged below 25).

The relative contribution of all STI care providers within a specific region - in terms of NG control, i.e. testing, diagnosis and identifying the characteristics of their patient populations - has not yet been comprehensively assessed. It has been estimated that, in England, GPs diagnosed between 6% and 9% of NG cases between 2000 and 2011, when data from both GPs and genitourinary medicine (GUM) clinics was taken into account [8]. Based on sentinel data from Dutch GPs (2007), two thirds of STI-related episodes (not specifically NG) were seen by GPs versus one third by STI clinics [9]. Recently, we showed that GPs (~40%), STI clinics (~30%) and gynaecologists (~30%) accounted for almost all CT tests performed by regular STI care providers within a particular Dutch region [10].

All current Dutch STI guidelines recommend the use of vaginal swabs for women and first-void urine for men in order to detect urogenital NG. Among asymptomatic individuals, the GP guidelines state that NG testing should only be considered when the patient belongs to one of the following ‘risk groups’: MSM, (persons visiting) prostitutes, persons originating from STI endemic countries, persons with frequently changing sexual contacts (≥3 contacts in the 6 months prior to the patient visit) and persons with a partner belonging to one of these risk groups [11]. STI clinics serve high-risk groups and, hence, routinely perform urogenital NG tests irrespective of symptoms [12]. Screening of asymptomatic individuals is not mentioned in the guidelines for gynaecologists [13].

Regarding extragenital NG testing, GP guidelines recommend additional anorectal NG testing in the case of anal sex and/or anal symptoms and/or MSM, and additional oropharyngeal testing for prostitutes, MSM, and everyone with oral sex and oropharyngeal symptoms. For STI clinics, oropharyngeal and anorectal testing is routinely advised for prostitutes and needs to be considered after unprotected oral and anal sex, respectively, for heterosexual women. Because of the high prevalence of extragenital NG, oropharyngeal and anorectal testing is routinely advised for MSM. With respect to guidelines for gynaecologists, oropharyngeal and/or anorectal NG testing should be performed in the case of exposure (passive anal and/or oral sex) and/or symptoms, respectively, for men as well as women.

With respect to NG test practice, Dutch STI clinic guidelines recently (2015) recommended re-testing within 4–6 months after a positive NG test, with a maximum interval of 1 year, in order to detect new infections, because of a high NG prevalence among men and women previously treated for NG [11]. Following a NG positive test, the Centers for Disease Control and Prevention (CDC) recommends re-testing after 3 months, and no later than 12 months [1]. On the other hand, Dutch GPs and gynaecologists have a re-testing policy based on risk behaviour [12, 13]. This means that a re-test should be performed when the patient is likely re-exposed to an untreated person with NG (the source). The optimal testing interval after such exposure is 3 weeks, and nucleic acid amplification tests (NAAT) are the first-choice tests followed by culture.

Increasing antimicrobial resistance has resulted in international guidelines including the recommendation that a test-of-cure (TOC) is performed 2 weeks after the completion of NG treatment (for NAAT) [5, 14]. Indeed, mathematical modelling has suggested that the most effective control strategy for the treatment of resistant NG is to follow-up infections that have already been treated in order to assess treatment failures, rather than only testing (and treating) more patients [15]. Current Dutch STI clinic, GP and gynaecologist guidelines recommend a TOC when the first-choice antibiotic (ceftriaxone) was not given and when symptoms persist or recur [11–13]. In addition, Dutch GPs should perform a TOC in pregnant women (4–6 weeks after treatment) [12] and STI clinic guidelines advise a TOC in the case of oropharyngeal NG because it is more difficult to eradicate than urogenital and anorectal NG, and is frequently asymptomatic [11].

In combination, the testing and subsequent adequate treatment of positives is an essential tool for NG control and needs to be optimised in order to reduce NG transmission, NG-related morbidity and the chance of increasing antibiotic resistance among NG strains.

Here, we compared NG diagnostic testing practices between the different STI care providers (GPs, an STI clinic and gynaecologists) from one defined geographic Dutch region. We also assessed whether NG positives were re-tested within 3–12 months in order to detect a new infection, and if TOC (within 3 months after treatment) was applied following NG treatment. We hope that our results will help to improve NG testing protocols for different STI care providers.

## Methods

### Study population

Data sources used to identify all NG tests administered by the STI clinic, GPs and gynaecologists from January 2006 to August 2010 were set up in such a way that each row represented one patient visit (during which ≥1 NG test was taken). Per visit, a maximum of three NG tests could be taken, depending on the anatomical locations tested/sampled (i.e. urogenital, anorectal and oropharyngeal). Data were retrieved from our own public health STI clinic medical records (*n* = 28,459 tests from 21,570 patient visits) and from the Department of Medical Microbiology (Zuyderland Medical Centre) serving both GPs and gynaecologists (*n* = 9214 tests from 9204 patient visits). These data covered a nearly complete (>95%) region in the southern part of the Netherlands (Parkstad, eastern South Limburg) [10]. In the study area one STI clinic is present and approximately 130 GPs and 7 gynaecologists are providing their care to the inhabitants.

**Fig. 1:**
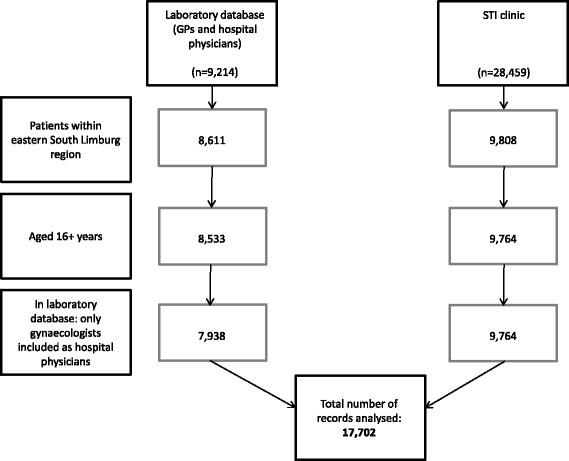
Selection procedure for the records analysed per database. Note. GP, general practitioner; STI, sexually transmitted infection

All data sources provided age, sex, and 4-digit postal code of the tested person, as well as date, anatomic location and result of the test. Using postal code and age, records were selected of patients aged 16+ years living in the specified region. Hospital physicians belonged to 14 different specialties, predominantly gynaecology (77.4%). Aside from gynaecology, dermatology (*n* = 365) and urology (*n* = 169) were the specialties that performed most NG tests. In order to reduce heterogeneity between hospital physicians, tests by non-gynaecology medical specialties - i.e. 594 urogenital and 1 anorectal NG tests - were excluded from analyses. These represented 3.3% (*n* = 595) of all NG tests, including 4 urogenital NG positives. The final dataset for analyses therefore included: 17,702 tests from 15,458 patient visits: STI clinic (*n* = 9764 tests from 7528 patient visits), GPs (*n* = 5903 tests from 5902 patient visits) and gynaecologists (*n* = 2035 tests from 2028 patient visits) (Fig. 1).

CT tests and diagnoses were retrieved from a dataset with the same selection criteria, which resulted in a total of 31,647 CT tests from 29,366 visits [10]. All patients who were tested for NG, were tested for CT as well.

Social economic status (SES) scores were extracted per postal code area and are based on national data on income, educational level and employment (see below).

### NG diagnosis

STI clinic specimens mainly comprised self-collected vaginal swabs and urine. Clinician-collected urethral and cervical swabs were predominantly used by GPs and gynaecologists. Anorectal (mainly self-collected) and oropharyngeal (provider-collected) swabs were used for testing these respective anatomical locations. Strand displacement amplification (SDA) and polymerase chain reaction (PCR) for NG testing (Becton Dickinson ProbeTec ET system, Maryland, USA and from 6 to 1-2010 Abbott M2000, Illinois, USA) were used for the evaluation of all specimens. In our study population, no NG diagnosis was made by culture.

### Statistical analyses

First, a descriptive analysis was performed to assess the contribution of each STI care provider regarding the number of patient visits and positives diagnosed. Patient visits during which NG tests were taken that turned out positive were defined as ‘test positive visits’. NG positivity refers to the proportion of test positive visits with the denominator being the total number of patient visits.

Second, to assess the factors associated with 1) the number of patient visits and 2) test positive visits, multivariable Poisson and logistic regression analyses were performed, respectively, including age (16–21, 22–24, 25–29, 30–39 and 40+), SES (low, middle and high, based on tertiles), and test calendar year (continuous). Moreover, a concurrent (i.e. at the same visit) CT test positive result was included as a factor in the analyses of the test positive visits. In the evaluation of the number of patient visits, differences between providers (GP, STI clinic and gynaecologists) were evaluated (the same comparisons could not reliably be carried out for the test positive visits due to low numbers). Denominator data for all subgroups included in Poisson regression analyses were retrieved from Statistics Netherlands (http://www.cbs.nl; age, sex and test year) and the Netherlands Institute for Social Research (http://www.scp.nl; SES) [10].

The multivariable Poisson analysis was initially performed including ‘provider’ and any potential confounders (age, test year and SES) to test for differences in outcomes between STI care providers. Subsequent analyses to determine associations between the outcomes and age, test year and SES were stratified by STI care provider, because of statistically significant interaction-terms between ‘provider’ and the other factors.

All analyses were stratified by sex, due to interactions. The outcome measures presented are adjusted for age, test year, a concurrent CT test positive result and SES (where appropriate).

Third, the proportion of NG positive patients who were re-tested was compared across providers. A re-test was defined as a NG test taken between 3 and 12 months after the NG positive test visit, in accordance with the CDC guidelines [1]. Moreover, NG positivity was assessed among the re-tests per STI care provider. In addition, patient characteristics of patients who were re-tested were compared with those who were not re-tested by means of binary logistic regression analyses.

Finally, TOC was evaluated for the different STI care providers in a similar way as described above for the re-tests. TOC was defined as a NG test taken between 2 weeks and 3 months after the NG positive test visit [14].

For both re-test and TOC only the first positive test of a patient was included in the analyses. Tests taken within 2 weeks from this first positive test (*n* = 7) were excluded from the TOC and re-test analyses.

Analyses were performed using SPSS version 20 (IBM Corp. Released 2011. IBM SPSS Statistics for Windows, Version 20.0. Armonk, NY: IBM Corp.). A *P*-value <0.05 was considered statistically significant.

## Results

### NG testing practices

In total, 11,459 individuals contributed to 17,702 NG tests that were performed during 15,458 patient visits. These included 15,445 urogenital, 1416 anorectal and 841 oropharyngeal tests. The number of NG tests per STI care provider were: STI clinic (*n* = 9764), GPs (*n* = 5903) and gynaecologists (*n* = 2035). In total, 811 anorectal tests were performed in men and 605 in women. Oropharyngeal tests were performed in 521 men and in 320 women. Extragenital tests were almost exclusively (99%, *n* = 2243) performed at the STI clinic.

### Contribution of different STI care providers in testing

**Fig. 2:**
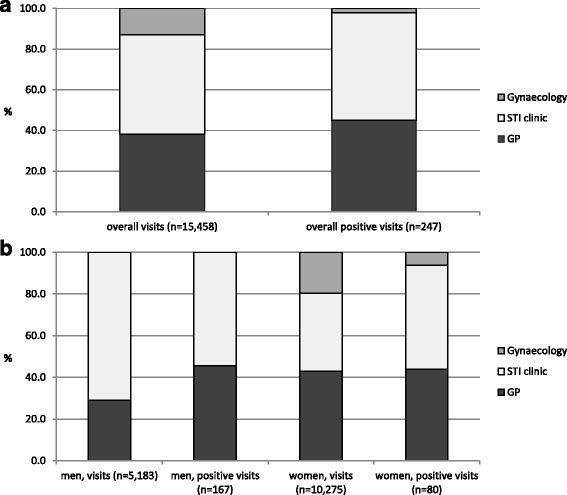
Contribution of the STI care providers in terms of number of patient visits during which *Neisseria gonorrhoeae* tests were taken (i.e. visits) and positives diagnosed (i.e. positive visits), overall (**a**) and by sex (**b**). Note. GP, general practitioner; STI, sexually transmitted infection

Overall, the STI clinic accounted for 49% (*n* = 7528) of all patient visits, whereas GPs and gynaecologists accounted for 38% (*n* = 5902) and 13% (*n* = 2028), respectively (Fig. 2a and Table 1). Men were mostly tested by the STI clinic (71% of men), while women were more often tested by their GPs (43% of women, Fig. 2b and Table 1).

**Table 1:** Evaluation of the effects of STI care provider, age, time and social economic status on the number of *Neisseria gonorrhoea* tests, irrespective of anatomical location^a^

	N per	GP		STI clinic		Gynaecology	
	category	(*N* = 5902)		(*N* = 7528)		(*N* = 2028)	
		N	Adj. RR (95% CI)	N	Adj. RR (95% CI)	n	Adj. RR (95% CI)
Men							
Provider	5183	1499	1 (ref)**	3684	2.26 (1.81–2.81)	n.a.	n.a.
Age							
16–21 years	467	144	1 (ref)**	323	1 (ref)**	n.a.	n.a.
22–24 years	796	178	2.37 (1.45–3.86)	618	3.72 (2.32–5.96)	n.a.	n.a.
25–29 years	1590	401	3.15 (1.96–5.07)	1189	4.67 (2.93–7.46)	n.a.	n.a.
30–39 years	998	346	1.25 (0.76–2.08)	652	1.30 (0.82–2.09)		
40+ years	1332	430	0.36 (0.22–0.59)	902	0.65 (0.39–1.08)		
Test year (cont.)^b^			1.30 (1.17–1.44)**		1.10 (0.99–1.23)	n.a.	n.a.
SES^c^						n.a.	n.a.
low	1658	597	1.57 (1.08–2.27)	1061	1.10 (0.75–1.60)	n.a.	n.a.
medium	1802	461	1.02 (0.71–1.46)	1341	1.11 (0.77–1.61)	n.a.	n.a.
high	1507	435	1 (ref)**	1072	1 (ref)	n.a.	n.a.
Women			2.99 (2.42–3.70)^d^		1.19 (0.95–1.48)^d^	n.a.	n.a.
Provider	10,275	4403	1 (ref)**	3844	1.05 (0.85–1.30)	2028	0.50 (0.39–0.65)
Age							
16–21 years	1591	711	1 (ref)**	659	1 (ref)**	221	1 (ref)**
22–24 years	1551	571	1.84 (1.24–2.73)	759	2.45 (1.61–3.72)	221	2.07 (1.06–4.07)
25–29 years	2932	1209	2.34 (1.57–3.49)	1128	2.21 (1.43–3.42)	595	3.24 (1.60–6.57)
30–39 years	2082	973	0.88 (0.59–1.33)	591	0.64 (0.42–0.97)	518	1.36 (0.69–2.72)
40+ years	2119	939	0.19 (0.13–0.29)	707	0.24 (0.14–0.41)	473	0.28 (0.15–0.51)
Test year (cont.)^b^			1.24 (1.13–1.36)**		1.09 (0.98–1.23)		1.98 (1.64–2.38)**
SES^c^							
low	3581	1684	1.44 (1.03–2.00)	1066	1.12 (0.78–1.60)	831	1.73 (0.99–3.03)
medium	3541	1423	1.05 (0.75–1.47)	1514	1.28 (0.89–1.84)	604	1.05 (0.58–1.90)
high	2883	1271	1 (ref)**	1024	1 (ref)	588	1 (ref)

*Adj. RR* adjusted rate ratio, *CI* confidence interval, *GP* general practitioner, *STI* sexually transmitted infection, *SES* social economic status, *CT* *Chlamydia trachomatis*

Overall *P*-values for categorical variables were specified by: *P < 0.05 **P < 0.01

^a^Calculated by means of multivariable Poisson regression analysis, including provider, age, test year and SES, where appropriate. The rows stating ‘Provider’ should be read horizontally and include the analyses of differences in number of tests between providers. Analyses evaluating differences in number of tests by age, test year and SES were stratified per provider and should be read vertically

^b^Range of test years: 2006–2010

^c^The numbers given for the SES categories do not add up to the total number of tests performed, because of missing SES data for a proportion of patients

^d^ Comparison of *Neisseria gonorrhoea* testing rates between men and women; men are reference category

After adjusting for age, test year and SES, the STI clinic performed most tests in men, whereas both the STI clinic and GPs performed more tests than the gynaecologists in women (Table 1).

### NG positivity

Taking into account all 15,458 patient visits, 247 (1.6%) resulted in at least one NG positive test. Proportion of test positive visits per STI care provider were: GP 1.9% (*n* = 111), STI clinic 1.7% (*n* = 131) and gynaecology 0.2% (*n* = 5). Stratified by anatomical location NG positivity was: urogenital (1.2%, *n* = 191), anorectal (3.3%, *n* = 47) and oropharyngeal (4.5%, *n* = 38). Visits resulting in positive NG tests from more than one anatomical location were only observed at the STI clinic. In 73% (77.8% in men and 59.1% in women) of the extragenital NG infections, no urogenital NG was diagnosed concurrently.

The urogenital NG positivity in men was 2.4% (123/5177) and 0.7% (68/10,268) in women. For extragenital NG, these numbers were: 4.8% (39/811) in men and 1.3% (8/605) in women (anorectal); and 4.6% (24/521) in men and 4.4% (14/320) in women (oropharyngeal).

### Contribution of different STI care providers in NG positivity

Overall, GPs accounted for 45% (*n* = 111), the STI clinic for 53% (*n* = 131), and gynaecologists for 2.0% (*n* = 5) of all test positive visits (Fig. 2a). Both men and women had most test positive visits at the STI clinic (55% of visits from men and 50% of visits from women, Fig. 2b).

After adjusting for STI care provider, age, test year and SES, NG positivity in men was higher at the GP than at the STI clinic, and in women NG positivity was higher at the GP and STI clinic than at the gynaecologist (Table 2). Concurrently testing CT positive was associated with NG positivity in both men and women (Table 2), and this was observed across all providers.

**Table 2:** Evaluation of the effects of STI care provider, age, time and social economic status on the number of *Neisseria gonorrhoea* positives, irrespective of anatomical location^a^

	Test positivity per category		Men Test positivity 3.2% (167/5183)		Women Test positivity 0.8% (80/10,275)	
	N	%	n	Adj. OR (95% CI)	n	Adj. OR (95% CI)
Provider						
GP	111	1.9	76	1 (ref)**	35	1 (ref)*
STI clinic	131	1.7	91	0.48 (0.35–0.66)	40	1.33 (0.83–2.12)
Gynaecologist	5	0.2	n.a.	n.a.	5	0.35 (0.14–0.92)
Age						
16–21 years	33	1.6	21	1 (ref)**	12	1 (ref)
22–24 years	38	1.6	23	0.63 (0.35–1.16)	15	1.23 (0.57–2.64)
25–29 years	50	1.1	32	0.41 (0.23–0.72)	18	0.84 (0.40–1.76)
30–39 years	53	1.7	39	0.78 (0.45–1.36)	14	1.08 (0.49–2.37)
40+ years	73	2.1	52	0.84 (0.50–1.42)	21	1.58 (0.76–3.26)
Test year (cont.)^b^				0.86 (0.76–0.97)*		0.89 (0.76–1.05)
SES^c^						
low	90	1.7	59	1.09 (0.74–1.62)	31	1.17 (0.67–2.02)
medium	80	1.5	54	0.99 (0.66–1.48)	26	0.92 (0.52–1.62)
high	68	1.5	46	1 (ref)	22	1 (ref)
CT positive	42	3.2	29	1.92 (1.26–2.92)**	13	2.21 (1.19–4.09)**

*Adj. OR* adjusted odds ratio, *CI* confidence interval, *GP* general practitioner, *STI* sexually transmitted infection, *SES* social economic status, *CT* *Chlamydia trachomatis*

Overall *P*-values for categorical variables were specified by: * *P < 0.05* ** *P* < 0.01

^a^Calculated by means of multivariable logistic regression analysis, including provider, age, test year and SES

^b^Range of test years: 2006–2010

^c^The numbers given for the SES categories do not add up to the total number of tests performed, because of missing SES data for a proportion of patients

### NG re-test

Of the 177 urogenital test positive visits, 36 (20.3%) were followed by a re-test. Stratified by STI care providers, this proportion was: GP 12.7% (13/102), STI clinic 32.9% (23/70) and gynaecology 0% (0/5). For extragenital NG (only diagnosed at the STI clinic) the proportion followed by a re-test was: 43.6% (17/39) for anorectal NG and 57.1% (20/35) for oropharyngeal NG.

Overall, re-test positivity was 16.9% (10/59), stratified by anatomical location: urogenital 13.9% (*n* = 5; three at the GP and two at the STI clinic), anorectal 17.6% (3/17) and oropharyngeal 10.0% (2/20).

Patients aged 40+ were re-tested more frequently than 16–21 year olds, and a higher proportion of patients with high SES were re-tested compared with low SES patients (Table 3).

**Table 3:** Overview of patient characteristics based on whether a person was re-tested or not after a positive NG test

	Re-tested		
	Yes (*N* = 59)	No (*N* = 166)	
	n (%)^a^	n (%)^a^	OR (95% CI)
Sex			
Men	35 (23.5)	114 (76.5)	1 (ref)
Women	24 (31.6)	52 (68.4)	1.5 (0.8–2.8)
Age			
16–21	5 (16.7)	25 (83.3)	1 (ref)
22–24	2 (5.9)	32 (94.1)	0.3 (0.1–1.7)
25–29	14 (30.4)	32 (69.6)	2.2 (0.7–6.9)
30–39	12 (25.5)	35 (74.5)	1.7 (0.5–5.5)
40+	26 (38.2)	42 (61.8)	3.1 (1.1–9.1)*
SES^b^			
low	15 (18.3)	67 (81.7)	1 (ref)
mid	17 (22.7)	58 (77.3)	1.3 (0.6–2.9)
high	25 (42.4)	34 (57.6)	3.3 (1.5–7.0)*
CT test result			
negative	51 (27.3)	136 (72.7)	1 (ref)
positive	8 (21.1)	30 (78.9)	0.7 (0.3–1.7)

*OR* odds ratio, *CI* confidence interval, *SES* social economic status, *CT Chlamydia trachomatis*

*P < 0.05

^a^Denominator of the percentages given is the total number of patients per row

^b^The numbers given for the SES categories do not add up to the total numbers per category, because of missing SES data for a proportion of patients

### Test-of-cure

A TOC was performed after 18 of 177 (10.2%) urogenital NG test positive visits. Per STI care provider, these numbers were: GP 11.8% (12/102), STI clinic 5.7% (4/70) and gynaecology 20.0% (2/5). Positive TOC for urogenital NG was only observed at the GP (overall 16.7%, *n* = 2).

Regarding extragenital NG, the proportion of TOC performed was 17.9% (7/39) for anorectal NG and 17.1% (6/35) for oropharyngeal NG. Two positive TOC were observed in relation to anorectal, and one in relation to oropharyngeal locations. These positive extragenital TOCs were all performed more than 2 months after the initial positive test.

## Discussion

This comprehensive overview of NG test practices within a defined Dutch geographical region shows that most men were tested at the STI clinic and most women were tested at their GP. Interestingly, hardly any extragenital NG tests were performed outside the STI clinic. Most NG positives were detected by GPs or at the STI clinic, while hardly any cases were observed in the hospital setting. Moreover, approximately 10% of the patients testing NG positive at the GP were re-tested for a new infection (after 3 to 12 months), whereas this proportion was ~50% at the STI clinic for extragenital NG. TOC was performed in less than 20% of cases irrespective of STI care provider and anatomical location.

A strength of this study is that we utilised a comprehensive dataset revealing the NG test practice of all main regular STI care providers serving residents of one geographical region. By using unique anonymised codes, we could follow-up NG positive patients and evaluate the frequency/occurrence of both re-testing and TOC. Moreover, the data included information about the anatomical testing site, enabling the evaluation of both urogenital and extragenital NG testing procedures. Finally, NG re-testing practices across three STI care providers were described in detail, which provided valuable information regarding both timing and the patient populations who were re-tested.

One limitation of our study is that we did not have access to more detailed information regarding the characteristics (e.g. sexual orientation) of the individuals tested, which made it impossible to compare NG test practice between e.g. heterosexual men and MSM. Based on the results of a previous Dutch study, we can infer that ~10% of men with a STI-related GP visit are MSM [16].

In addition, we could not evaluate whether TOC was implemented according to current Dutch TOC guidelines, because of missing information regarding pregnancy status, whether or not symptoms were persistent, and which antibiotics were prescribed to NG positive patients [11, 12].

Finally, the time intervals chosen for re-tests and TOC is somewhat debatable. In this study, we have used internationally accepted intervals for re-testing (3–12 months after a test positive visit) and TOC (2 weeks after treatment) [1, 14].

This study is the first of its kind to provide more precise estimates regarding the relative contribution of STI care providers to NG testing in the Netherlands [9]. The STI clinic had the highest contribution to the number of NG tests performed, which was mainly explained by the fact that almost three out of four NG tests in men were performed at the STI clinic. For women, a more equal distribution was observed between GPs and STI clinic, whereas gynaecologists contributed ~20% of all NG tests in women. The fact that men are less likely than women to visit a GP regarding STI-related symptoms has been addressed previously [17].

As well as testing (and treating) men who present with symptoms indicative of NG, GPs need to be aware of the sexual preference of their male patients. In this way, testing can be targeted towards their MSM patients, a well-known group at-risk for NG [1]. In addition to urogenital testing, and in light of the widely observed high proportion of single site extragenital NG [3, 11], MSM should also be tested for NG both anorectally and oropharyngeally. Importantly, these extragenital sites have been implicated as drivers for NG antimicrobial resistance [18]. The current STI guidelines (2013) for Dutch GPs recommends that extragenital tests should be performed on indication, and this will hopefully be translated into clinical practice [12].

In this perspective, GPs can also draw attention to the possibility of NG self-testing, for example, by providing information leaflets and posters in their practice, because self-collected extragenital NG swabs have been found to be a feasible, valid and acceptible alternative for MSM and women [19].

Most NG tests were performed among patients aged 25–29, irrespective of sex and provider, whereas patients aged 40+ received comparatively fewer tests. This is in line with previous Dutch data in which STI consultations by GPs were evaluated and the highest proportion of patients tested were in their twenties [16]. GP and gynaecology patients with low SES were more likely to be tested than those with high SES. This association was not observed at the STI clinic, a finding which has previously been explained by the selection of high-risk groups at these clinics which effectively dilutes any effects of SES [10].

We observed that, overall, one in five urogenital NG positive tests were followed by a re-test. More specifically, 35% of urogenital NG positives were re-tested at the STI clinic, while a lower proportion of positives were re-tested at the GP surgery or by a gynaecologist (0–10%). With regard to extragenital NG testing at the STI clinic, this proportion was higher (40–50% were re-tested). It is known that persons who test NG positive have a higher a priori chance of testing NG positive again (~10%) [1]. Although our numbers are small, our data also confirms this. In the Netherlands, STI clinics have already adopted the recommendation to re-test between 4 and 6 months after the initial test positive visit [11]. It may be worthwhile for GPs and gynaecologists to include the recommendation for NG re-testing in their guidelines.

With respect to the patient characteristics of person who were re-tested, we observed that older patients with higher SES were more likely to receive a re-test. Future studies could address this evaluation in more depth, for example by including the sexual orientation of patients, to make firm conclusions regarding patient groups that are mainly re-tested.

TOC was performed in ~10% of cases following a urogenital test positive visit, with GPs and gynaecologists performing the test relatively more often, although numbers for gynaecologists were small. High prescription rates of ciprofloxacin by Dutch GPs could explain the higher proportion of TOC performed by the GPs [20].

In relation to extragenital NG, TOC was performed in 15–20% of cases. Several guidelines, including the European guidelines on the diagnosis and treatment of NG in adults and the ones for Dutch STI clinics, have stated that TOC should always follow an oropharyngeal NG treatment, because oropharyngeal NG is more difficult to eradicate than urogenital and anorectal NG, and is frequently asymptomatic [11, 14]. Until now, no ceftriaxone resistance has been detected in the Netherlands [21]. Because this resistance is most likely to emerge in the groups at highest risk for NG, the implementation of this policy is particularly important for patients presenting at the STI clinic. In this respect, the proportion of TOC performed following oropharyngeal NG at the STI clinic (~17%) was low in our study, and needs to be addressed. The 3 positive extragenital TOCs observed in our study, were all performed at the STI clinic more than 2 months after the initial positive test, thus more likely reflecting re-exposure, rather than being an indication of ceftriaxon resistance.

Moreover, STI care providers need to be aware of the timing of TOC by NAAT, as several of those reported here were performed too early, i.e. within a week of the test positive visit, therefore compromising the result (as this can be still based on detection of non-viable nucleic acids after treatment of the NG infection) [22].

## Conclusions

Our study of NG test practice showed that, despite the fact that NG can present at anorectal and oropharyngeal sites, often without a concurrent urogenital infection, general practitioners and gynaecologists rarely test for extragenital NG, and, as a result, a substantial amount of treatment opportunities are missed. In addition, re-testing yields relatively high positivity rates and, therefore, STI care practitioners should consider NG re-testing to detect new infections following initial NG diagnosis. Finally, it showed that laboratory data on NG testing are useful in order to evaluate and optimise NG test practice.

## Abbreviations

CDC: Centers for Disease Control and Prevention
CI: Confidence interval
CT: *Chlamydia trachomatis*
GP: General practitioner
GUM: Genitourinary medicine
HIV: Human immunodeficiency virus
MSM: Men who have sex with men
NAAT: Nucleic acid amplification test
NG: *Neisseria gonorrhoeae*
OR: Odds ratio
PCR: Polymerase chain reaction
RR: Rate ratio
SDA: Strand displacement amplification
SES: Social economic status
STI: Sexually transmitted infection
TOC: Test-of-cure
